# PReS-FINAL-2327: Abatacept in the treatment of refractory uveitis

**DOI:** 10.1186/1546-0096-11-S2-P317

**Published:** 2013-12-05

**Authors:** D Moretti, T Giani, G Vannucci, E Marrani, G Simonini, I Pagnini, R Cimaz

**Affiliations:** 1 Anna Meyer Children Hospital, Florence, Italy

## Introduction

Uveitis is one of the most serious manifestations of Juvenile Idiopathic Arthritis (JIA), with the potential to cause severe sight-threatening ocular complications. The first line treatment consists of topical and oral steroids with DMARDs therapy for more severe cases, including immunosuppressive and anti-TNFα agents. Abatacept (CTLA-4 immunoglobulin) is a soluble, fully human fusion protein that consists of the extracellular domain of CTLA-4, linked to a modified Fc portion of the human immunoglobulin IgG1, which does not activate the complement. The drug is approved for the treatment of polyarticular JIA.

## Objectives

We evaluated the efficacy and safety of Abatacept in a series of young patients with sight-threatening JIA-related and idiopathic uveitis refractory to previous anti-TNFα agents.

## Methods

We performed a monocenter collection of data of 12 patients affected by uveitis (10 JIA-related and 2 idiopathic) which resulted refractory to both classic immunosuppressive and anti-TNFα treatments and who started a therapy with Abatacept at a monthly dosage of 10 mg/kg, administered intravenously. Abatacept was administered as off-label use in 7/12 patients (4 with oligoarticular JIA, 1 with enthesitis-related arthritis JIA and 2 with idiopathic uveitis). Nine patients reached a 6 months observation period, while the other three have been followed for only 2 months. Side effects, mean frequency of uveitis flares and ocular complications before and after treatment were recorded.

## Results

Abatacept treatment led to sustained improvement of ocular manifestations in all patients. Results after the 6 months observation period showed a decrease in the mean frequency of uveitis flares, which decreased from 1.66 to 0.55 (-64.75%) during the 6 months before and 6 months after treatment. In those patients who have still not reached the 6 months observation period the administration of Abatacept led to a severe reduction of the mean frequency of uveitis which passed from 3.33 episodes during the two months before the administration to 0.33 after Abatacept introduction. No adverse effects were observed. No new ocular complications or worsening of preexisting ones were reported.

## Conclusion

Our experience with Abatacept resulted positive in terms of safety and efficacy. The introduction of Abatacept led to severe improvement of ocular manifestations in both refractory JIA-related and idiopathic uveitis. Our results confirm those of the current literature and postulate the role of Abatacept as a promising alternative treatment in refractory uveitis.

## Disclosure of interest

None declared.

